# Remote follow-up after cataract surgery (CORE-RCT): study protocol of a randomized controlled trial

**DOI:** 10.1186/s12886-023-02779-7

**Published:** 2023-01-30

**Authors:** Janneau L. J. Claessens, Joukje C. Wanten, Noël J. C. Bauer, Rudy M. M. A. Nuijts, Oliver Findl, Josef Huemer, Saskia M. Imhof, Robert P. L. Wisse

**Affiliations:** 1 Department of Ophthalmology, University Medical Center Utrecht, Postbus 85500, Huispostnummer E03.136, Utrecht, 3508 GA the Netherlands; 2 University Eye Clinic Maastricht, Maastricht University Medical Center+, Maastricht, the Netherlands; 3 Vienna Institute for Research in Ocular Surgery, a Karl Landsteiner Institute, Hanusch Hospital, Vienna, Austria; 4 Moorfields Eye Hospital NHS Foundation Trust, London, UK

**Keywords:** Cataract, E-health, Telemedicine, Remote care, Eye care, Easee

## Abstract

**Background:**

Cataract surgery has become one of the most performed surgical procedures worldwide. Postoperative management consists of routine clinical examinations to assess post-operative visual function and detect possible adverse events. Due to the low incidence of complications, the majority of clinic visits after cataract surgery are uneventful. Nonetheless, valuable time and hospital resources are consumed. We hypothesize that remote post-operative follow-up involving teleconsultations and self-assessments of visual function and health status, could be a valid alternative to face-to-face clinical examinations in selected patient groups. The practice of remote follow-up after cataract surgery has not yet been evaluated. The aim of this study is to investigate the validity, safety and cost-effectiveness of remote cataract surgery follow-up, and to report on the patients’ experiences with remotely self-assessing visual function.

**Methods:**

This study is a multicenter, open-label, randomized controlled trial. Patients planned for cataract surgery on both eyes, without ocular comorbidities, are eligible for participation. Participants will be allocated (1:1) into one of the two study groups: ‘telemonitoring’ or ‘usual care’. Participants in the ‘telemonitoring’ group will perform in-home assessments after cataract surgery (remote web-based eye exams and digital questionnaires on their own devices). Participants in the ‘usual care’ group will have regular post-operative consultations, according to the study site’s regular practice. Outcome measures include accuracy of the web-based eye exam for assessing visual acuity and refraction, patient-reported outcome measures (visual function and quality of life), adverse events, and cost aspects.

**Discussion:**

Investigating remote follow-up after cataract surgery fits the current trends of digitization of health care. We believe that remote self-care can be a promising avenue to comply with the increasing demands of cataract care. This randomized controlled trial provides scientific evidence on this unmet need and delivers the desired insights on (cost)effectiveness of remote follow-up after cataract surgery.

**Trial registration:**

ClinicalTrials.gov: NCT04809402. Date of registration: March 22, 2021.

**Supplementary Information:**

The online version contains supplementary material available at 10.1186/s12886-023-02779-7.

## Background

Cataract, clouding of the eye’s natural lens, is one of the leading causes of blindness and visual impairment worldwide [1, 2]. It is most commonly age-related, and therefore a common condition amongst older-aged adults [2]. The main treatment is surgical extraction of the cataract, followed by implantation of a clear artificial lens [3]. Across the European Union member states, cataract surgery is conducted 4.3 million times yearly, making it the most performed surgical procedure [4]. Due to increasing life expectancies and aging of the population, these numbers are expected to keep increasing.

Over the last decades, new technologies and surgical techniques have revolutionized the procedure, making cataract extraction one of the safest surgeries to be performed [2, 5]. Based on the latest report of the European Registry of Quality Outcomes for Cataract and Refractive Surgery (EUREQUO), 98% of the procedures remained uneventful [6]. Infectious endophthalmitis, the most dreaded short-term post-operative complication with a devastating prognosis, only occurred in 0.01% of the cases [6]. Other short-term post-operative consultations include cystoid macular edema (0.46%), persistent corneal edema (0.02%), and uncontrolled elevated intraocular pressure (0.02%) [6].

Cataract surgery is usually performed in day care. A recent innovation is immediate sequential bilateral cataract surgery, further improving the cost-effectiveness of cataract care as both eyes will be operated on the same day [7]. Typical postoperative follow-up consists of a short-term evaluation within a few days after surgery and a long-term evaluation approximately 1 month after surgery [8]. The main purpose of the short-term postoperative consultation is to ascertain no complications have occurred immediately after surgery, such as an elevated intraocular pressure [9–12]. At approximately 1 month after surgery, the residual refractive error is determined and routine postoperative cataract care is finished. Due to the low rate of (serious) adverse events, the majority of postoperative examinations after cataract surgery will be uneventful [5–14]. Nevertheless, because of the high number of cataract surgeries performed, postoperative follow-up of cataract patients takes up a considerable amount of hospital time and resources. To maximize the efficiency of postoperative cataract care, the clinical examination shortly after surgery is often replaced by a telephone consultation [15]. Notwithstanding, telephone consultations lack quantifiable outcome parameters.

Remote follow-up could be a cost-effective and patient friendly alternative to conventional in-hospital follow-up. A remote monitoring platform that includes assessments of visual function using e-health technology could enable patients to self-monitor their postoperative eye status and remotely provide quantifiable outcome parameters, such as visual acuity, to their eye care professional.

An e-health tool that can be used for this purpose has been developed by the Amsterdam-based medtech company Easee (https://easee.online). It allows users to self-assess their visual acuity and refractive error via a website, using their own electronic devices (a computer or tablet, and a smartphone). Previous research indicated the refraction assessment of this tool to be non-inferior to a manifest refraction performed by an eye care professional, investigated amongst healthy volunteers with refractive errors, achieving the best outcomes in low myopes [16]. Moreover, studies have shown that visual acuity can be reliably assessed in patients with various ocular conditions [16–18]. Most studies have been targeting relatively younger-aged individuals and we suspect that introducing e-health technology to older-aged generations will be more challenging. Interestingly, a recent study on the performance of the remote assessment in post-operative cataract patients identified that the majority of patients were able to complete the assessment and achieve accurate visual acuity scores [19]. This study was performed in a supervised, controlled setting and only evaluated distance visual acuity assessments. In the present study, the remote exam will be more comprehensive and also include refractive error assessments, as well as near vision. Moreover, examinations will be performed unsupervised by patients at home, using their own smartphones and computers, mimicking a future real-world application of this tool.

In summary, we hypothesize that remote follow-up could be a valid alternative to conventional face-to-face examinations in post-operative cataract care. This randomized controlled trial aims to investigate the validity, safety and cost-effectiveness of remote follow-up after cataract surgery, and provide insights on patients’ experiences with remotely assessing visual function.

## Methods

### Study design and setting

This study will assess the validity of a web-based eye exam, report on patients’ experiences with this tool, and evaluate the cost-effectiveness and safety of remote follow-up. Therefore, we will perform a randomized controlled trial to make a comparison between two different methods of post-operative follow-up: “telemonitoring” vs. “usual care”. The study is titled the CORE-RCT: Cataract Online Refraction Evaluation – a Randomized Controlled Trial. The protocol for this study was designed according to the SPIRIT 2013 guidelines [20].

Multiple centers will participate in the trial. The sponsor of this study is the University Medical Center Utrecht, the Netherlands. Participating centers are the Maastricht University Medical Center+, the Netherlands; Amphia Hospital Breda, the Netherlands; Oogcentrum Noordholland, the Netherlands; Vienna Institute for Research in Ocular Surgery, Austria; and Augenklinik Sulzbach, Germany. Each of the participating sites reviewed a copy of the research protocol and provided written approval and agreement to participate in this study. The study has been approved by the Medisch Ethische Toetsingscommissie Utrecht, the Netherlands (NL74625.041.21); the Ethikkommission der Stadt Wien, Austria (EK 20-334-0121); and the Ethikkommission Saarbrücken, Germany (Ha 44/18).

### Study objectives

This randomized controlled trial aims to evaluate remote follow-up after cataract surgery. The objectives of this trial can be categorized into four categories: validity, safety, cost-effectiveness, and patients’ experiences.

#### Validity of the web-based eye exam

Validity of the web-based eye exam (developed by Easee B.V.) will be assessed by comparing the web-based outcomes to the clinical findings at the visit scheduled 4-6 weeks after surgery. Our main objective will be to determine if the corrected distance visual acuity (CDVA) achieved with the refraction resulting from the remotely performed web-based refraction assessment is non-inferior to the achieved CDVA with the prescription of the in-hospital manifest refraction. A mean difference between the two scores up to 0.10 logMAR (one ETDRS line) will be considered clinically acceptable (i.e. non-inferior). In addition, we will assess uncorrected distance visual acuity (UDVA) and compare the outcomes of the web-based vs. the in-hospital assessment. Furthermore, we will evaluate the accuracy of the web-based exam for determining refractive error and uncorrected near visual acuity, by comparing these outcomes to the conventional in-hospital assessments. The repeatability of the web-based refractive assessment will be determined by comparing the outcomes 4-6 weeks after surgery to the outcomes 3 months after surgery (on the condition that post-operative complications resulting in a change of visual function are absent).

#### Safety of remote follow-up

Safety will be evaluated by reporting on the occurrence of (serious) adverse events in both groups. Furthermore we will evaluate to what extent the digital triage questionnaires can detect alarming symptoms and adverse events.

#### Cost-effectiveness of remote follow-up

The cost-effectiveness will be evaluated by quality-adjusted life years (QALYs), total costs (societal and hospital costs), and the probability of adverse events or additional clinical examinations. The main outcome measure will be incremental cost-effectiveness ratio (ICER), defined as euros per QALY (based on the EQ5D-5 L questionnaire [21]), and compared between the two study groups.

#### Patients’ perspective

Firstly, we will determine if remotely self-assessing vision influences patient-reported outcome measures of visual function (Catquest-9SF and NEI-VFQ-25 questionnaires [22, 23]) and quality of life (EQ-5D-5L questionnaire [24]), by comparing the outcomes between both randomization groups.

Secondly, we will evaluate the user experiences with the web-based tool by a custom quantitative questionnaire, distributed amongst the participants of the telemonitoring group at the end of the study. This questionnaire is based on the theoretical Technology Acceptance Model; a commonly used model to evaluate and incorporate user experiences in the development process of technology. Since its introduction by Davis in 1989 [25], the model has been extended by multiple research teams, including for utilization in health care settings [26–28]. We used the extended models as a reference framework and developed a study-specific questionnaire in cooperation with the University of Twente, the Netherlands (Additional file 1). Lastly, the quantitative results are enriched with in-depth qualitative interviews (Additional file 2). Dutch-speaking participants of the telemonitoring group will be invited to report on their experiences with the web-based eye exam. Interviews will be conducted by researchers experienced in qualitative interview studies, until data saturation is reached (i.e. when no longer new insights are gained).

### Study population and sample size calculation

Patients planned for bilateral cataract surgery without visual acuity influencing comorbidities are eligible for study participation. The surgical procedures can be performed on the same day (i.e. immediate sequential) or on two different days. Exclusion criteria are: cataract surgeries combined with other procedures (including keratoplasty, vitrectomy, glaucoma filter implants), presence of ocular comorbidities that negatively influence post-operative visual acuity (such as amblyopia, age-related macular degeneration, diabetic retinopathy, glaucoma or uveitis), insufficient command of the Dutch, German or English language, no access to a smartphone and computer/tablet, and inability to successfully perform the demo version of the web-based eye exam.

The sample size calculation is based on determining the validity of the web-based eye exam since calculations based on cost-effectiveness and safety were not feasible. We aim to assess whether the corrected distance visual acuity obtained with the web-based refraction is not significantly worse than the visual acuity obtained with the manifest refraction. We assume no difference between the measurements and consider a difference up to 0.10 logMAR to be non-inferior. With a standard deviation of 0.30 logMAR (a commonly used SD in power calculations on visual acuity [29–31]), an α of 0.05, a power of 90, 20% loss to follow-up and using a one-sided, one sample t-test, 94 eyes are then required in the telemonitoring group (so 47 participants, as all measurements will be performed bilaterally). This results in a total study population of 94 participants (188 eyes) for both study groups.

### Study procedures

**Fig. 1:**
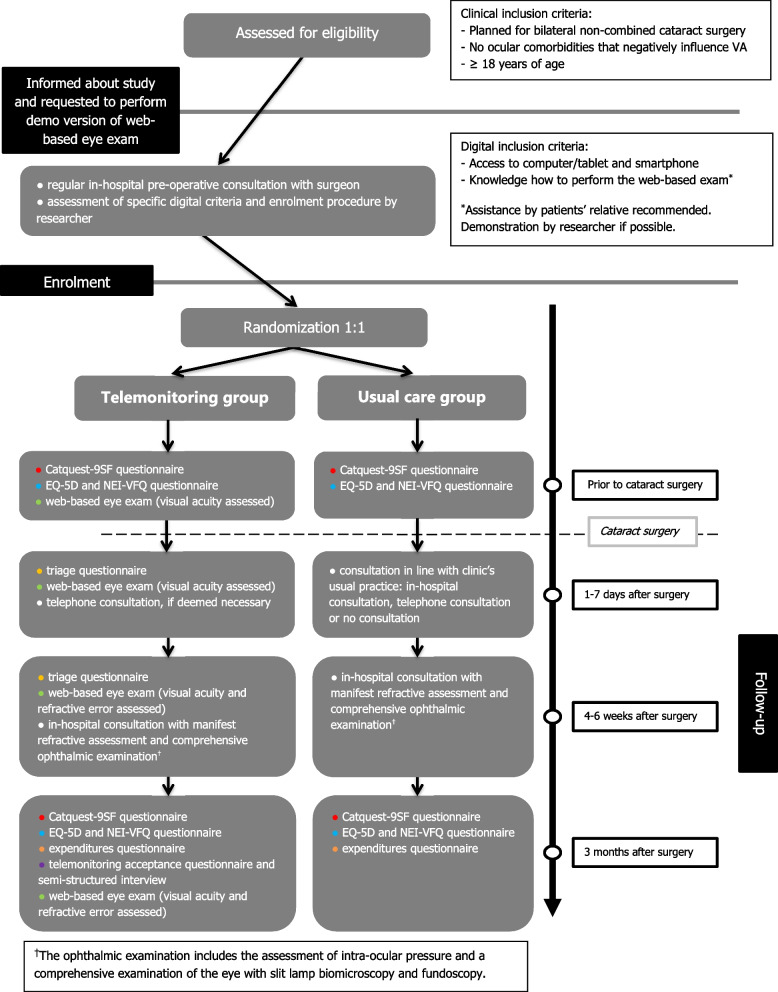
Flowchart of the CORE-RCT study procedures. Overview of study procedures: recruitment, randomization and study measurements

A flowchart describing the study procedures is depicted in Fig. 1.

#### Recruitment and informed consent procedure

Eligible patients will be identified by the ophthalmologist or delegated personnel at the outpatient department, when cataract is diagnosed and the cataract surgery will be planned for both eyes. All eligible patients will be provided with written information about the study. Patients who are interested to participate, will be invited to perform a demo version of the web-based eye exam at home. We always recommend assistance by a relative. The demo contains the home set-up phase and a shortened test flow, and aims to assure that the technical requirements and a sufficient level of digital proficiency for study participation are met, preventing a high loss-to-follow-up after enrolment. Patients who fail to access the demo version of the web-based eye exam, will be excluded from randomization. To report on this participation bias, we will keep track of an overview of all invited patients and record reasons for non-participating.

If all inclusion criteria are met and the patient is willing to participate, written informed consent will be obtained. After enrolment, participants can leave the study at any time for any reason if they wish to do so. Participants who have not completed the questionnaires and/or performed the web-based eye exam prior to surgery (i.e. at baseline) will be withdrawn from the study and replaced.

#### Randomization

Participants will be randomized 1:1 using a computer-generated block size permuted randomization list (block sizes 2 and 4). The randomization will be stratified for treatment center and age (< 69 and ≥ 69 years). The age stratification is based on prior experiences with the remote monitoring platform regarding age-related digital literacy, and the distribution of cataract incidence among age deciles (mean age in the Netherlands in 2019 was 73 years [14]).

#### Study measurements

The ‘telemonitoring’ group will have a post-operative follow-up involving web-based eye exams, digital questionnaires and telephone consultations. The invitations for the web-based eye exams and questionnaires will be sent via e-mail at 4 specific time points: prior to surgery, < 1 week after the surgery, 4-6 weeks after the surgery and 3 months after surgery. Prior to surgery, visual acuity will be assessed with the participant’s current spectacles, if applicable. After surgery, the web-based eye exam will be performed without spectacles. In addition, participants will fill out triage questionnaires to identify any (alarming) symptoms.

Shortly (1-7 days) after surgery, uncorrected distance visual acuity (UDVA) will be assessed remotely, and a telephone consultation will take place if deemed necessary by the surgeon. Approximately 1 month (i.e. 4-6 weeks) after surgery, the web-based tool will assess uncorrected visual acuity (distance and near) and the residual refractive error. After performing this remote assessment at home, participants will have an in-hospital ophthalmic examination with conventional visual acuity and refraction assessments for validity and safety purposes. The observer and participant will be blinded for the web-based refraction outcome during the manifest refraction. At this in-hospital consultation, distance visual acuity will be assessed by an ETDRS chart at 4 m, both uncorrected (UDVA) and corrected (CDVA). The CDVA will be assessed twice: using both the prescriptions of the web-based and the manifest refraction. Near visual acuity will be assessed uncorrected using a Sloan ETDRS chart at 40 cm. Three months after surgery, the web-based assessment will be repeated and participants will be requested to fill out a questionnaire about their experiences with the web-based eye exam. In addition, a sample of the Dutch-speaking participants will be interviewed to further explore their perspective on remote follow-up after cataract surgery.

The ‘usual care’ group will have a post-operative follow-up adhering to the clinic’s usual practice. Typically, regular consultations will be planned within 1 week after surgery (i.e. short-term evaluation) and at approximately 4-6 weeks after surgery (i.e. long-term evaluation). The latter will include a full ophthalmic examination and refraction assessment, to assess post-operative visual outcomes and residual refractive errors. In some of the participating centres, the short-term follow-up evaluation will be a telephone consultation instead of an in-hospital consultation, or no consultation at all, depending on the standard guidelines of this clinic.

For both study groups, all adverse events and additional consultations - at other moments than the specified time points in the study flow chart - will be registered. Prior to (i.e. at baseline) and 3 months after surgery, participants of both groups will be requested to fill out questionnaires about quality of life (EQ-5D-5L [24]) and visual function (Catquest-9SF [22], NEI-VFQ-25 [23]). Three months after surgery, all participants will be requested to fill out a short custom questionnaire about expenditures related to hospital visits (such as costs for transportation and parking).

### Statistical analysis

Data will be collected and analyzed preoperative (i.e. at baseline) and at three postoperative time points. All quantitative variables will be summarized. The data will be tested for distribution, and if not normally distributed the corresponding non-parametric test will be used. For all analyses, a *P* < .05 is considered statistically significant.

Comparisons between the web-based eye exam and the conventional clinical assessments will be analysed according to the Bland-Altman methodology [32]. We will compare assessments of distance visual acuity, near visual acuity and refractive error. Furthermore, we will analyse independent associations between clinical characteristics and agreement between the web-based exam and the conventional reference tests. Safety of the remote follow-up will be evaluated by comparing the occurrence of adverse events in both study groups. The cost-effectiveness will be assessed by QALYs, total costs, and the probability of adverse events and additional clinical examinations.

## Discussion

This is the first study to investigate remote follow-up after cataract surgery including refractive assessments. We believe that remote follow-up fits in the current trends of digitization of health care, and that it can be a promising avenue to tackle the increasing demands of cataract care. The studied population, i.e. cataract patients, is particularly interesting due to the high volume of procedures and low risks. We are aiming to provide a clear overview on validity, safety, cost aspects, and patients’ perspectives regarding remote follow-up after cataract surgery. After completion of the present study, the web-based eye exam test flow will be improved and an iteration in algorithm development will take place, focusing on additional training, recalibrating, and a machine learning approach to better control user-behavior and -environment. We anticipate to amend the current trial with a telemonitoring-only approach to further explore the performance and deliver fine granular data on the (cost-)effectiveness of the updated web-based tool.

## Supplementary Information

**Additional file 1.** Study-specific ‘telemonitoring acceptance’ questionnaire.

**Additional file 2.** Interview topic list.

## Data Availability

Data sharing is not applicable to this article as it does not contain any data.

## Abbreviations

CORE-RCT: Cataract Online Refraction Evaluation - a Randomized Controlled Trial
CDVA: Corrected distance visual acuity
UDVA: Uncorrected distance visual acuity
QALY: Quality-adjusted life years
