# Supplementary material for: Remote follow-up after cataract surgery (CORE-RCT): study protocol of a randomized controlled trial
Source: BMC Ophthalmol. 2023 Jan 30;23:41. doi: 10.1186/s12886-023-02779-7 (PMC9885558; doi:10.1186/s12886-023-02779-7)
Supplement: Supplementary file 1 — Additional file 1. Study-specific ‘telemonitoring acceptance’ questionnaire. [file 12886_2023_2779_MOESM1_ESM.docx]

## Additional File 1

##### Study-specific ‘telemonitoring acceptance’ questionnaire

| **Behavioral Intention**   1. Overall, I am highly willing to use e-health services like the web-based eye test in the future 2. Before using it for the first time, I was curious about the web-based eye test. 3. I find it would be good to use a web-based eye test to monitor vision after surgery. |
| --- |
| **Performance Expectancy**   1. I find a web-based eye test useful. 2. The web-based eye test helps me to monitor my vision. |
| **Effort Expectancy**   1. The instructions of the web-based eye test are clear and understandable. 2. It was easy for me to learn how to use the web-based eye test. 3. I find the web-based eye test easy to use. 4. Using the web-based eye test does not require me much effort. |
| **Facilitating Conditions**   1. Before participating in the study, I had the resources (smartphone, computer or tablet) available to do the web-based eye test. 2. I feel like I have the knowledge necessary to fully complete the web-based eye test. 3. I believe there is adequate assistance available when encountering problems with the web-based eye test. |
| **Technology Anxiety**   1. I feel sufficiently skilled to use a computer or smartphone for e-health services like the web-based eye test. 2. I hesitate to use a computer or smartphone (for e-health services like the web-based eye test) for fear of making mistakes. 3. The web-based eye test is somewhat intimidating to me. 4. Using the web-based eye test makes me feel uncomfortable. |
| **Self-efficacy**   1. I could complete the web-based eye test if there was no one around to tell me what to do. 2. I need assistance by someone else while using the web-based eye test. |
| **Trust/reliability**   1. I feel I can trust the web-based eye test. 2. I trust in the data protection and privacy of e-health services like the web-based eye test. |
| **Doctor’s opinion**   1. I trust my doctor’s judgment. 2. I trust my doctor’s judgment about the use of the web-based eye test. |

Answer option: 5-point likert scale.

5 = strongly agree; 4 = agree; 3 = neither agree nor disagree; 2 = disagree; 1 = strongly disagree
