# Supplementary material for: Remote follow-up after cataract surgery (CORE-RCT): study protocol of a randomized controlled trial
Source: BMC Ophthalmol. 2023 Jan 30;23:41. doi: 10.1186/s12886-023-02779-7 (PMC9885558; doi:10.1186/s12886-023-02779-7)
Supplement: Supplementary file 2 — Additional file 2. Interview topic list. [file 12886_2023_2779_MOESM2_ESM.docx]

## Additional File 2

##### Interview topic list

*Note:* *The original interview guide was in Dutch. It has been translated to English for publication only.*

General experience

1. How did you experience the web-based eye test?
2. Did the web-based eye test meet your expectations? Are there things that surprised you in a positive or negative way?

User friendliness

1. Did you find the test easy to use? Why or why not?
2. Were you able to understand the instructions well? Why or why not?
3. Did you see the results of the test? Were you able to understand it / how did you interpret it? Did it raise any questions?
4. (If Q5 = yes: Do you like the fact that a result is presented immediately? Why or why not? Would you be happy to receive the test result without explanation from a doctor?)
5. How could the test be improved?

External influences

*Environment and social influences*

1. Where did you perform the web-based eye test? How did it go? How long did it take?
2. Did you have any assistance while performing the test at home? From whom? (If not: Why not?)

*Intention to use in the future*

1. Would you use the web-based eye test again if needed? Why or why not?
2. How would you feel about the last in-hospital consultation being replaced by a remote eye assessment (and a phone appointment)?
